# Non-infectious uveitis affecting the posterior segment treated with fluocinolone acetonide intravitreal implant: 3-year fellow eye analysis

**DOI:** 10.1038/s41433-021-01608-9

**Published:** 2021-06-11

**Authors:** Carlos Pavesio, Carsten Heinz

**Affiliations:** 1 Moorfields Eye Hospital NHS Foundation Trust, London, UK; 2 NIHR Biomedical Research Centre at Moorfields Eye Hospital NHS Foundation Trust and UCL Institute of Ophthalmology, London, UK; 3 Department of Ophthalmology, St. Franziskus Hospital Muenster, Muenster, Germany; 4 Department of Ophthalmology, University Duisburg-Essen, Essen, Germany

**Keywords:** Retinal diseases, Education

## Abstract

**Background:**

Prevention of non-infectious uveitis of the posterior segment (NIU-PS) recurrence using 0.2 μg/day fluocinolone acetonide implant (FAi) was assessed over 3 years (NCT01694186). Outcomes for FAi-treated and fellow eyes with NIU-PS were compared, to evaluate FAi versus conventional treatment strategies.

**Methods:**

Eligible subjects had >1-year recurrent NIU-PS history and either ≥2 separate recurrences requiring treatment, or corticosteroid therapy (systemic or ocular) in the 12 months preceding study entry. Bilateral disease was present and analysed in 59/87 FAi-treated participants. Recurrence rates, best-corrected visual acuity (BCVA) changes, cataract surgery, intraocular pressure (IOP) events and adjunctive medication use were compared for FAi-treated and fellow eyes.

**Results:**

Over 36 months, more FAi-treated than fellow eyes remained recurrence-free (28.8% vs. 5.1%, *P* = 0.001; mean 1.9 vs. 4.7 recurrences, respectively, *P* < 0.0001). FAi-treated eyes gained +9.6 letters BCVA, versus a loss of −4.4 in fellow eyes (*P* < 0.0001). Systemic medications were given to 42.4% of subjects. Intra/periocular adjunctive injections were lower in FAi-treated than fellow eyes (20.3% vs. 66.1%, *P* < 0.0001); topical corticosteroid use was also lower in FAi-treated than fellow eyes (27.1% vs 52.5%, *P* = 0.0041). IOP-related events occurred at similar rates in both FAi-treated and fellow eyes, excepting IOP-lowering surgery (5.1% vs. 15.3%, respectively; *P* = 0.1251). Cataract surgery occurred in 72.0% of FAi-treated and 37.0% of fellow eyes.

**Conclusions:**

In subjects with bilateral NIU-PS, continuous, low-dose corticosteroid with 0.2 μg/day FAi reduced recurrence and adjunctive medication requirements, and improved vision over 36 months, providing greater protection against ocular inflammation than a reactive approach using standard of care.

## Introduction

Non-infectious uveitis of the posterior segment (NIU-PS) is an inflammatory disease of the eye, which, if recurrent and untreated or unmanaged, can damage tissue and threaten vision as a result of repeated periods of inflammation [1]. NIU-PS is estimated to cause 10–15% of cases of blindness in the developed world [2] and there is a high prevalence of bilateral NIU-PS.

Treatment of NIU-PS using a 0.2 μg/day fluocinolone acetonide implant (FAi, ILUVIEN; Alimera Sciences Ltd., Aldershot, Hampshire) to prevent recurrence of ocular inflammation has previously demonstrated effectiveness in a 36-month study (Study 001; clinicaltrials.gov NCT01694186) [3]. The 0.2 μg/day FAi continuously releases a submicrogram dose of fluocinolone acetonide to the back of the eye over a 36-month period and based on Study 001 and other results [4], the FAi is indicated for prevention of relapse in NIU-PS of the eye [5].

Many patients enrolled in Study 001 had bilateral NIU-PS disease, but the bilateral outcomes were not reported [3]. These patients were studied in the current analysis to assess treatment outcomes in the same patient, where one eye received 0.2 μg/day FAi and the fellow eye was treated with the standard of care. The aim of the analysis was to compare the response in the FAi-treated eye with that of the fellow eye receiving conventional treatments and to better understand the progression of the disease over time.

## Subjects and methods

Details of this Phase 3, prospective, randomised, double-masked, multicentre comparison of 0.2 µg/day FAi-treated and control-treated (sham injection plus standard of care) subjects with NIU-PS have been previously described [3, 4]. Patients were eligible for study inclusion if they provided written informed consent, were ≥18 years of age, had a diagnosis of NIU-PS in at least one eye for at least 1 year and had ≥2 recurrences of uveitis requiring systemic corticosteroid or immunosuppressive treatment, or intraocular corticosteroids. Eligible patients needed to have received systemic treatment for a minimum of 3 months in the 12 months preceding study entry, or at least two intraocular corticosteroid treatments for the treatment of uveitis in the 12 months preceding study entry. Any patients with infectious aetiology or evidence of glaucoma/ocular hypertension at screening visit were excluded. The Institutional Review Board at each study centre approved the study, which was performed in accordance with the tenets of the Declaration of Helsinki.

The current analysis focuses only on patients in the 0.2 μg/day FAi treatment arm who had bilateral NIU-PS; of the 87 participants treated with the 0.2 μg/day FAi, 59 had bilateral disease. In these patients with bilateral uveitis, the FAi-treated eye was the more severely affected eye fitting the inclusion/exclusion criteria (i.e. the eye having suffered more recurrences in the previous year, or if equal, the eye having received more therapy in the previous year, or if equal, the eye having the worse visual acuity or if equal, the eye clinically judged to be the more severely affected eye). If the eyes were affected equally, the FAi-treated eye was the right eye. The better-seeing (fellow) eye was managed according to the discretion of the treating physician, using current standard of care, including systemic corticosteroids.

For both FAi-treated and fellow eyes, number of recurrences, best-corrected visual acuity (BCVA) and central retinal thickness (CRT) change from baseline, lens status, rate of cataract surgery (based on lens status at baseline) and intraocular pressure (IOP) events within 36 months were assessed, in addition to the use of adjunctive medications. Parameters were measured at baseline (i.e. BCVA, CRT, lens status, and IOP) and then throughout the 36-month period.

Recurrence was observed directly (by ≥15-letter decrease in visual acuity or ≥2-step increase in anterior chamber cells or vitreous haze) or imputed (based on any corticosteroid or immunosuppressant use, regardless of recurrence criteria, or based on missed ophthalmic assessments at any timepoint) as described previously in more detail [3] (Supplementary information).

Baseline characteristics, IOP-related events and cataract surgery rates were retrospectively analysed using primarily McNemar’s or paired Student’s two-tailed *t*-tests. Other tests, in addition to the statistical plan, were utilised to compare data and included the two-tailed Fisher’s exact tests and Student’s *t*-tests. Data are reported as mean ± standard deviation (SD) unless otherwise stated. Data are presented per patient eye.

## Results

Baseline demographics and patient characteristics for the 59 patients with bilateral NIU-PS are shown in Table 1. A majority of these patients were receiving some form of systemic treatment for uveitis control at baseline (corticosteroid, 32.2% (*n* = 19); non-steroidal immunosuppression (including conventional and/or biologic), 20.3% (*n* = 12); no treatment, 47.5% (*n* = 28)).

**Table 1:** Baseline demographics and patient characteristics.

		0.2 μg/day FAi-treated eye (*n* = 59)	Fellow eye (*n* = 59)	Fisher’s exact probability *P* two-sided test
Age, mean ± SD, years		48.5 ± 13.97		N/A
Sex, % (*n*)		61% (36) female, 39% (23) male		N/A
Vitreous haze grade, % eyes (n)	0/0.5+	52.5 (31)	84.7 (50)	0.0003
	1/2+	47.5 (28)	15.3 (9)	0.0003
	3/4+	0.0 (0)	0.0 (0)	>0.05
Anterior chamber cells grade, % eyes	0/0.5+	91.5 (54)	98.3 (58)	0.207
	1 / 2+	8.5 (5)	1.7 (1)	0.207
	3 / 4+	0.0 (0)	0.0 (0)	>0.05
BCVA, mean ± SD, ETDRS letters		65.3 ± 17.17	70.6 ± 18.87	0.1133^a^
IOP, mean ± SD, mmHg		13.4 ± 3.06	14.7 ± 4.26	0.0594^a^

*BCVA* best-corrected visual acuity, *ETDRS* Early Treatment Diabetic Retinopathy Study, *FAi* fluocinolone acetonide implant, *IOP* intraocular pressure, *N/A* not applicable.

^a^Student’s *t*-test.

### Recurrence of uveitis

**Fig. 1:**
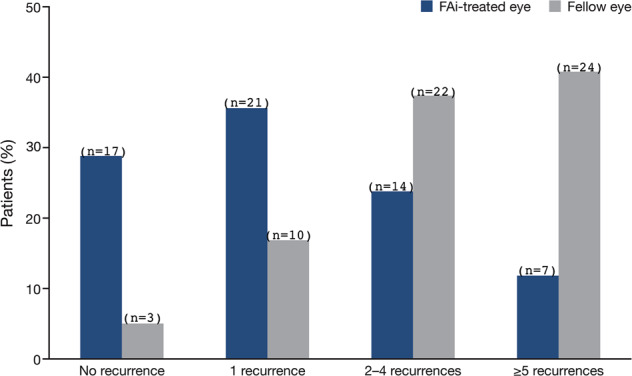
Proportion of patients having a cumulative number of recurrences at 36 months. The number of patient eyes per group is shown in parenthesis. The percentage of patients (*x*-axis) refers to the number of FAi or fellow eyes. FAi fluocinolone acetonide implant.

The FAi-treated eyes had a significantly reduced NIU-PS recurrence over 36 months of treatment compared with fellow eyes (71.2% (*n* = 42) of FAi-treated eyes had a recurrence (mean 1.9 ± 2.55 recurrences), compared with 94.9% (*n* = 56) of fellow eyes (mean 4.7 ± 3.97 recurrences; a difference of 2.8 ± 0.6 recurrences) (*P* < 0.0001, Student’s *t*-test). This equates to a difference of 23.7%, 95% confidence interval (95%) from 10.9 to 36.6, *P*=0.001, McNemar’s test (Fig. 1). The percentage of eyes with ≥2 recurrences over 36 months was substantially higher for fellow than for FAi-treated eyes (78.0% vs. 35.6% (*n* = 46 vs. 21), respectively; a difference of 42.4%, 95% CI 26.2–58.5; *P* < 0.0001, Fisher’s Exact test).

In further analysing these recurrence events (Supplementary information), a greater percentage of observed recurrences were recorded in fellow eyes (22.0%, *n* = 13) than in FAi-treated eyes (6.8%, *n* = 4) (a difference of 15.3%, 95% CI 2.9–27.6; *P* = 0.0336, Fisher’s Exact test); imputed recurrences were somewhat higher as well (72.9% vs. 64.4% (*n* = 43 vs. *n* = 38), respectively; a difference of 8.5%, 95% CI −8.2 to 25.1) (*P* = 0.4276, Fisher’s Exact test).

**Fig. 2:**
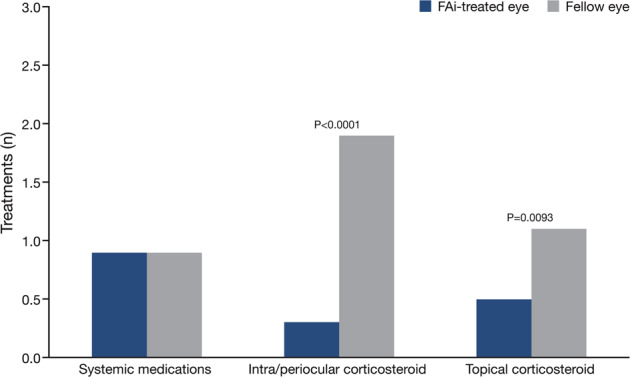
Mean cumulative number of adjunctive medications at 36 months. Mean data are based on the results from 59-eyes in both groups. FAi fluocinolone acetonide implant.

The percentage of eyes requiring adjunctive medication was 62.7% (*n* = 37) for the FAi-treated eyes compared with 94.9% (*n* = 56) for the fellow eyes (a difference of 32.2%, 95% CI 19.0–45.4; *P* < 0.001, McNemar’s test), with a mean of 1.7 ± 1.93 compared with 3.9 ± 3.19 treatments, respectively (a difference of 2.2, 95% CI 1.34–3.06; *P* < 0.001, Student’s *t*-test). In particular, the use of intra/periocular adjunctive corticosteroid injections was significantly lower in the FAi-treated eyes than in fellow eyes (20.3% (*n* = 12) vs. 66.1% (*n* = 39) (a difference of 45.8%, 95% CI 29.9–61.6; *P* < 0.0001, McNemar’s test). The mean for all 59 eyes in these groups was 0.3 ± 0.61 vs. 1.9 ± 2.37 treatments, respectively (*P* < 0.0001, Student’s *t*-test; Fig. 2). Use of topical corticosteroids was also significantly lower in the FAi-treated eye than in the fellow eye (27.1% (*n* = 16) vs. 52.5% (*n* = 31); a difference of 25.4%, 95% CI 8.4–42.5 (*P* = 0.0041, McNemar’s test). The mean number of treatments was 0.5 ± 0.92 vs. 1.1 ± 1.47, respectively (*P* = 0.0093, Student’s *t*-test; Fig. 2).

### Visual acuity outcomes

**Fig. 3:**
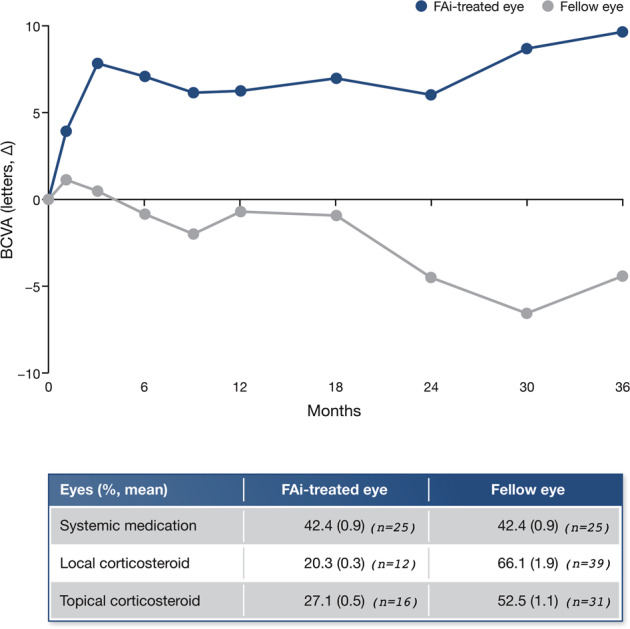
Change in BCVA and use of adjunctive medications. The number of patients presented in the graphic are: 59 (at time zero), 50 (month 1), 51 (month 3), 59 (month 6), 56 (month 9), 58 (month 12), 53/54 ((FAi-treated eye/fellow eye, respectively) month 18), 51 (month 24), 46 (month 30), and 49 (month 36). Numbers in the table below the graphic are shown in parenthesis with 59 patients being reported in each group. FAi fluocinolone acetonide implant. BCVA best-corrected visual acuity, FAi fluocinolone acetonide implant.

From Month 3 onwards and throughout 36 months, the mean BCVA in FAi-treated eyes stayed above 70 letters (6/12), while in the fellow eyes, vision declined from baseline and remained below 70 letters from Month 6 onwards (Supplementary Fig. 1). This was reflected in the change in BCVA over 36 months, with a significant difference in mean BCVA change between FAi-treated (9.6 ± 13.5 letters; *n* = 49) and fellow eyes (−4.4 ± 14.0 letters; *n* = 49) (a difference of 14.0 ± 2.8 letters; *P* < 0.0001, Student’s *t*-test) (see Fig. 3). These differences were observed despite the greater use of adjunctive treatment in the fellow eyes (Fig. 3).

The effect of therapy on visual acuity losses (defined as a decrease of ≥15 letters from baseline to Month 36) and the proportion of eyes with a VA of ≤33 ETDRS letters (i.e. 6/60 to 6/75 Snellen fraction) at baseline and Month 36 were assessed. In the FAi group, there were no patients with a loss of ≥15 letters versus 8 (16.3%) in the fellow eye (a difference of 16.3%, 95% CI 6.0–26.7; *P* = 0.0057, Fisher’s Exact test). In terms of VA ≤ 33 ETDRS letters, in the FAi group, there were five patient eyes (10.2%) at baseline and four (8.2%) in the fellow eye (a difference of 2.0, −9.4 to 13.5; *P* = 1.000, Fisher’s Exact test). This changed to 0 (0.0%) and 5 (10.2%), respectively, at Month 36 eye (a difference of 10.2, 1.7 to 18.7; *P* = 0.0562, Fisher’s Exact test).

### Control of oedema

Reduction in central foveal thickness occurred rapidly in the FAi-treated eyes and was maintained consistently through to Month 36; in the fellow eyes, there was poorer control generally and particularly between Months 18 and 30 (Supplementary Fig. 2).

### IOP, cataract, and adverse events

**Fig. 4:**
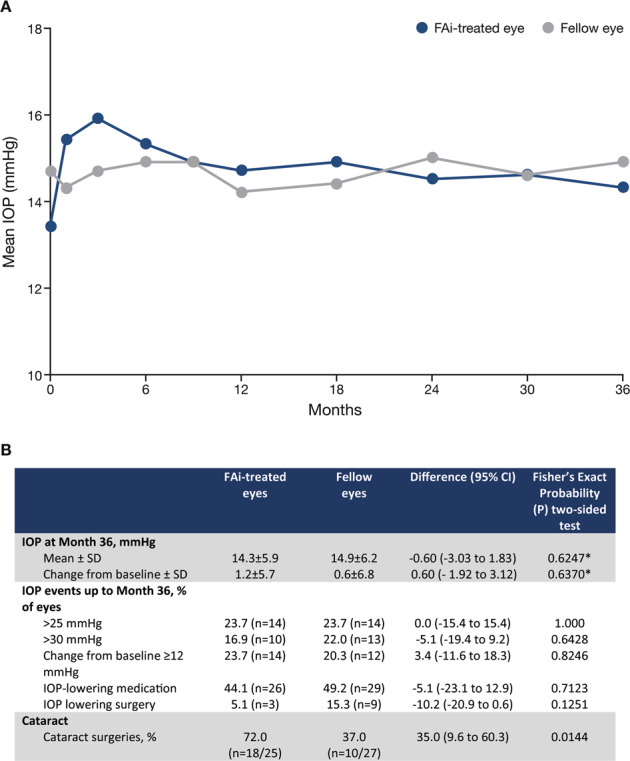
A IOP over 36 months’ follow up in both FAi-treated and fellow eyes. B IOP-related events over 36 months’ follow up in FAi-treated and fellow eyes. The number of patients presented in the graphic are: 59 (at time zero), 50 (month 1), 51/50 ((FAi-treated eye/fellow eye, respectively) month 3), 59/58 (month 6), 56 (month 9), 57 (month 12), 56 (month 18), 51 (month 24), 46 (month 30), and 49 (month 36). Numbers in the table below the graphic are from a population of 49 patients (mean and change in IOP at month 36) and 59 (for IOP events which are cumulative). Numbers for cataract surgeries are shown in parenthesis. FAi fluocinolone acetonide implant, IOP intraocular pressure.

Mean IOP levels remained in the normal range throughout the study (Fig. 4A) and IOP was well controlled in both FAi-treated and fellow eyes; most IOP elevations were treated with IOP-lowering medication (44.1% (*n* = 26) of FAi-treated eyes compared with 49.2% (*n* = 29) of fellow eyes (a difference of 5.1%, 95% CI −12.9 to 23.1; *P* = 0.7123, Fisher’s exact test) (Fig. 4B). However, the proportion of FAi-treated eyes receiving IOP-lowering surgery was lower than in the fellow eyes (5.1% (*n* = 3) vs. 15.3% (*n* = 9); a difference of 10.2%, 95% CI −0.6 to 20.9). However, this was not statistically different (*P* = 0.1251, Fisher’s exact test) and, like other comparisons for IOP events up to 36 months in Fig. 4B, did not reach statistical significance (*P* > 0.05, Fisher’s exact tests).

Cataract surgery was required more frequently over 36 months in the FAi-treated eyes than in the fellow eyes (72.0% (*n* = 18/25) vs. 37.0% (*n* = 10 of 27) of phakic eyes; a difference of 35.0%, 95% CI 9.6–60.3; *P* = 0.0144, Fisher’s exact test).

Serious treatment-emergent eye disorders were reported in 8 patient eyes (13.6%) in the FAi group and 14 eyes (23.7%) in the fellow eye. The eye disorders in the FAi group were primarily cataract (*n* = 4) and others included cystoid macular oedema, ocular hypertension, retinal detachment, uveitis and vitritis. In contrast, the eye disorders in the fellow eye were primarily reported as uveitis (*n* = 5), visual acuity loss (*n* = 3) and glaucoma (*n* = 3) and visual acuity loss (*n* = 2). Others included cataract, choroidal detachment, corneal epithelium defect, and cystoid macular oedema.

## Discussion

Patients with bilateral NIU-PS in the FAi arm of Study 001 were analysed over 36 months, with the fellow eyes acting as a comparative internal control for response to standard treatment and current treatment strategies. In real-world clinical practice, standard care typically entails a reactive approach, with local or systemic corticosteroid therapies given to address the inflammation, followed by improvement, resolution of inflammation and tapering of systemic treatment.

In the current study, more FAi-treated eyes remained recurrence free throughout 36 months than fellow eyes (28.8% vs. 5.1%); they achieved gains rather than losses in vision (9.6 letters vs. −4.4 letters, respectively; *P* < 0.0001) and had rapid and sustained reductions in central foveal thickness that were maintained for 36 months, despite less use of adjunctive medication (62.7% vs. 94.9%; *P* < 0.001). These results should be considered in the context of the FAi-treated eye being the one worst affected by NIU-PS, as the study was designed to actively manage the worst-seeing eye. The data show that inflammation was effectively managed with local therapy using the FAi, despite these eyes having worse posterior involvement at baseline.

NIU-PS is generally treated with corticosteroids; in severe cases, repeated intravitreal or systemic corticosteroid treatments may be required. This probably reflects two aspects of current care: the reactive management and subsequent tapering of systemic treatments leading to fluctuating levels of medication and therefore recurrent inflammation; and the necessary balance between a systemic dose that is effective and one that is tolerated. Repeated intravitreal and systemic treatments with corticosteroids can cause treatment-limiting adverse events such as IOP events requiring surgery, vitreous haemorrhage, or hyperglycaemia [6, 7]. Similarly, immunosuppressive drugs used to treat uveitis may have treatment-limiting adverse events. The medication levels required to control recurrences with systemic therapies may be too low to cross the blood–retinal barrier and treat ocular manifestations, while the levels required to achieve full control may be a lot higher and increase the incidence of systemic adverse events. While both eyes may have received a benefit from any systemic treatments administered, control was not achieved in the fellow eye; meanwhile, the FAi-treated eye received appropriate intraocular levels of corticosteroid that enabled effective control of recurrence with low-level systemic therapy. Consequently, a long-acting, continuous corticosteroid dosing option may offer an important alternative in these severe NIU-PS cases to the current standard of care.

FAi-treated eyes did require cataract surgery more often than fellow eyes (72.0% vs. 37.0%), but fewer FAi-treated eyes required IOP-lowering surgery than fellow eyes (5.1% vs 15.3%) with IOP well controlled in both FAi-treated and fellow eyes, primarily by using IOP-lowering medications. The reduction in corticosteroid treatment, both locally and topically, could explain the lower rate of IOP-lowering surgery in FAi-treated eyes than in fellow eyes, as elevated IOP, along with cataract formation, is a known adverse effect of corticosteroid treatment.

It is important to mention some of the limitations of the current analysis. Firstly, the types of uveitis (i.e. intermediate, posterior, and panuveitis) were not collected defined in the study and how results specifically apply to each of these is unclear. Furthermore, the study was a post hoc analysis of a previously reported study (PSV-FAI-001) and so the population was smaller than defined in the original study. Also, the original study was powered to detect changes at Month 6 as opposed to Month 36. Further, as a post hoc analysis, patients were not randomised, and so baseline characteristics were not completely balanced at baseline. Hence, there were small differences at baseline, which may have confounded the study findings. However, it is reassuring that differences were detected and the effect of the FAi versus the fellow eye was observed and favoured the FAi therapy. The current study had a relatively high number of recurrences that were imputed; however, whilst this may confound the results, data showed a separation between the treated and fellow eye in the same patients and this finding confirms the results of the study PSV-FAI-001 in terms of observed recurrences. Another consideration is that treatment of the fellow eye was not tied to a study protocol, so different strategies are likely and may also have contributed to the decline in BVCA over the 36-month period. Lastly, the study protocol did not quantitatively define the resolution of macular oedema.

In these subjects with bilateral NIU-PS, the collective outcomes presented here indicate that the continuous release of low-dose corticosteroid provided by the FAi gives greater protection against ocular inflammation and greater preservation of vision than a reactive approach using conventional strategies. In the current pandemic, this potential for an effective method to suppress intraocular inflammation over the long term, while reducing the need to expose patients to the risks of systemic immunosuppression, is a valuable treatment option. These long-term results comparing the effects of the FAi with the outcomes in the fellow eye treated with standard of care further extend our understanding of NIU-PS and show similar benefits to those demonstrated in the previous analysis of the 36-month data for the overall study population [3] indicating that clinical trials may find the fellow eye a good control when conducted in patients bilaterally affected by NIU-PS.

### Summary box

#### What was known before

1. Patients with NIU-PS are prone to recurrences, which, if not well-managed, can lead to long-term visual loss.
2. Current standard-of-care strategies frequently require reactive administration of systemic immunosuppressants or corticosteroids with subsequent tapering of medication once disease control is achieved, leading to cycles of remission and recurrence.
3. Treating patients with 0.2 μg/day FAi significantly improved outcomes compared with patients receiving standard of care.

#### What this study adds

1. By studying the fellow eye in patients with bilateral NIU-PS, this internal control enabled the clear demonstration of the benefit of FAi beyond any systemic treatment that was administered to manage the fellow eye.
2. Local administration of appropriate continuous, low-dose levels of corticosteroid can enable better control of NIU-PS inflammation with fewer adjunctive medications and potentially lower doses of systemic treatment.

## Supplementary information

Supplementary information

Supplementary Figure 1.

Supplementary Figure 2
