# Supplementary material for: Non-infectious uveitis affecting the posterior segment treated with fluocinolone acetonide intravitreal implant: 3-year fellow eye analysis
Source: Eye (Lond). 2021 Jun 11;36(6):1231–7. doi: 10.1038/s41433-021-01608-9 (PMC9151815; doi:10.1038/s41433-021-01608-9)

**Supplementary information**

- Recurrence was defined as:
- A ≥2-step increase in the number of cells in the anterior chamber (per high-powered field, 1.6× using a 1-mm beam), compared with any visit timepoint prior to Month 6, OR
- An increase in the vitreous haze of ≥2 steps, compared with any visit timepoint prior to Month 6, OR
- A deterioration in visual acuity of at least 15 letters best-corrected visual acuity (BCVA), compared with any visit timepoint prior to Month 6
- In addition, recurrence was imputed in the following cases:
- A subject who had not previously experienced a recurrence and did not complete the required eye examinations at Month 36 for any reason
- A subject who had not previously experienced a recurrence and took a systemic concomitant medication or a local concomitant medication in the study eye at any time during the study prior to Month 36
- In each instance, the cause of the change must have been attributable only to non-infectious uveitis. Further, a recurrence event was imputed if, for a previously non-recurrent study eye, the study eye was treated with a local or systemic medication, or the participant had a missing ophthalmic assessment at the 6-, 12- or 36-month visit. Medications were defined as follows: (1) oral, systemic, injectable or topical corticosteroids; or (2) systemic immunosuppressants

**Supplementary Figure 1. Mean BCVA over time**

The number of patients presented in the graphic are: 59 (at time zero), 50 (month 1), 51 (month 3), 59 (month 6), 56 (month 9), 58 (month 12), 53/54 ((FAi-treated eye/fellow eye respectively) month 18), 51 (month 24), 46 (month 30) and 49 (month 36). Abbreviations: BCVA, best-corrected visual acuity; FAi, fluocinolone acetonide implant
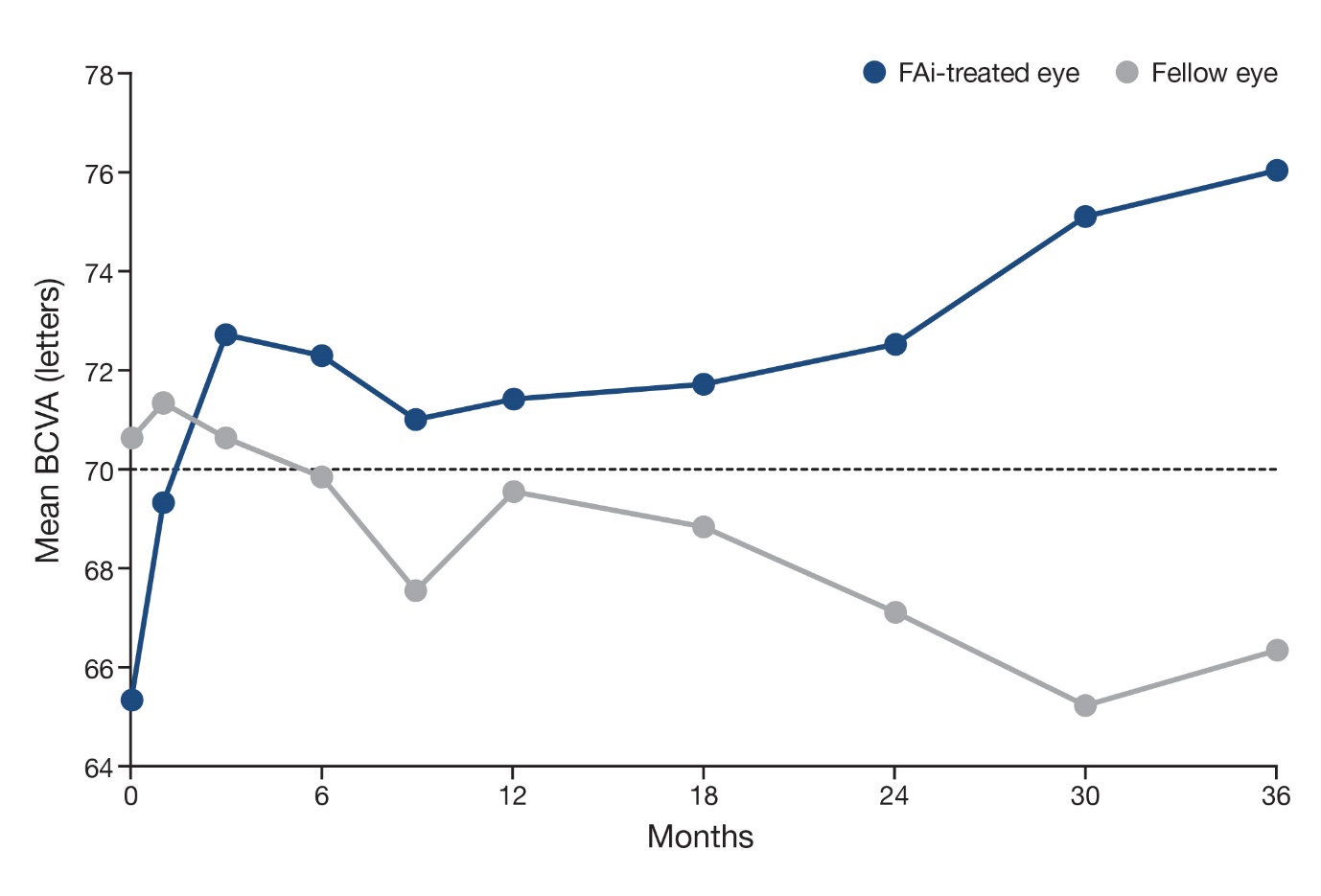


**Supplementary Figure 2. Mean retinal thickness (μm) over time**

The number of patients presented in the graphic are: 57/58 ((FAi-treated eye/fellow eye respectively) at time zero), 49/47 (month 1), 51 (month 3), 59/58 (month 6), 56/55 (month 9), 58 (month 12), 53/52 (month 18), 50 (month 24), 45/43 (month 30) and 49/48 (month 36). Abbreviation: FAi, fluocinolone acetonide implant.


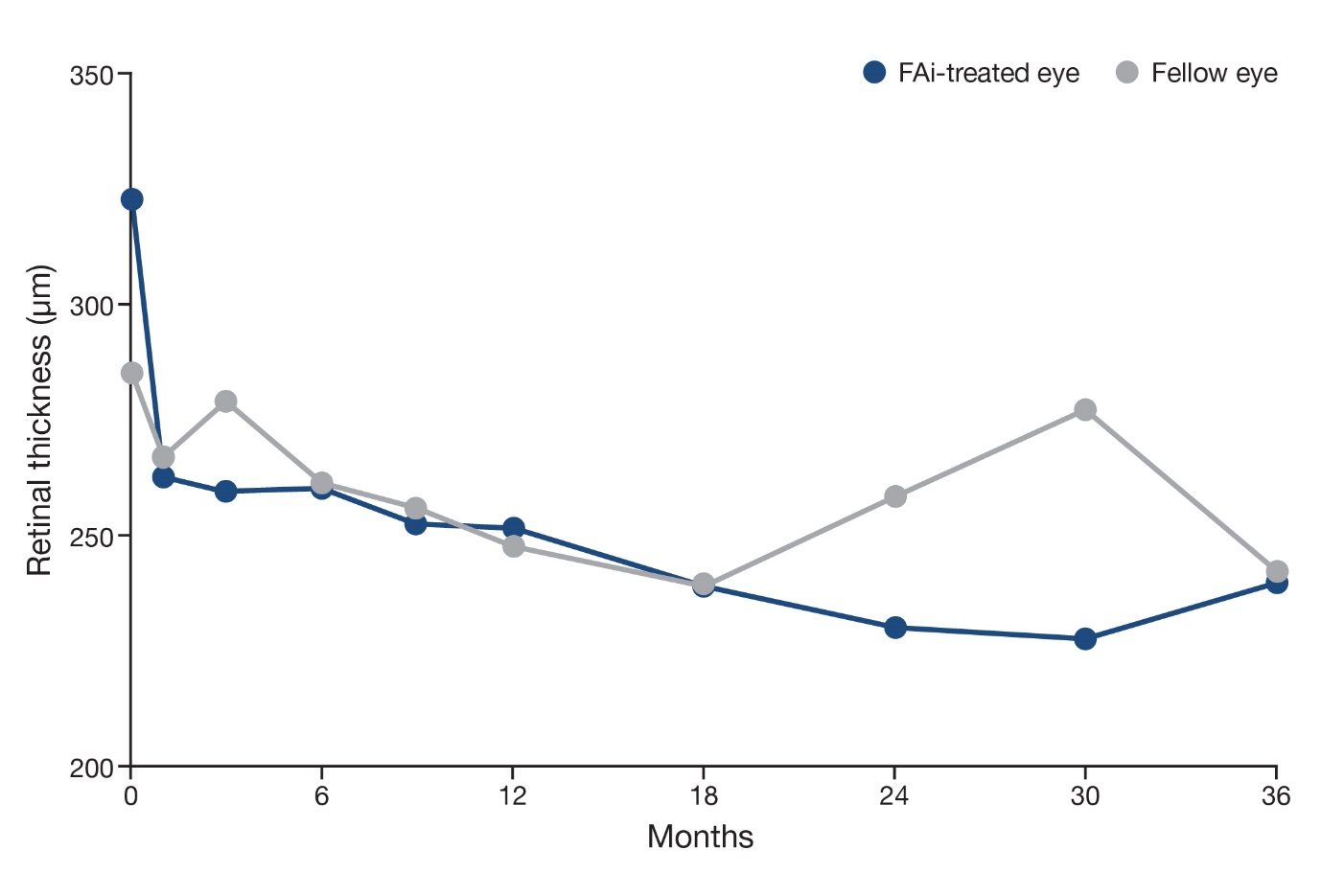

Supplement: Supplementary file 1 — Supplementary information [file 41433_2021_1608_MOESM1_ESM.docx]
