# Supplementary figures and images for: Non-infectious uveitis affecting the posterior segment treated with fluocinolone acetonide intravitreal implant: 3-year fellow eye analysis
Source: Eye (Lond). 2021 Jun 11;36(6):1231–7. doi: 10.1038/s41433-021-01608-9 (PMC9151815; doi:10.1038/s41433-021-01608-9)

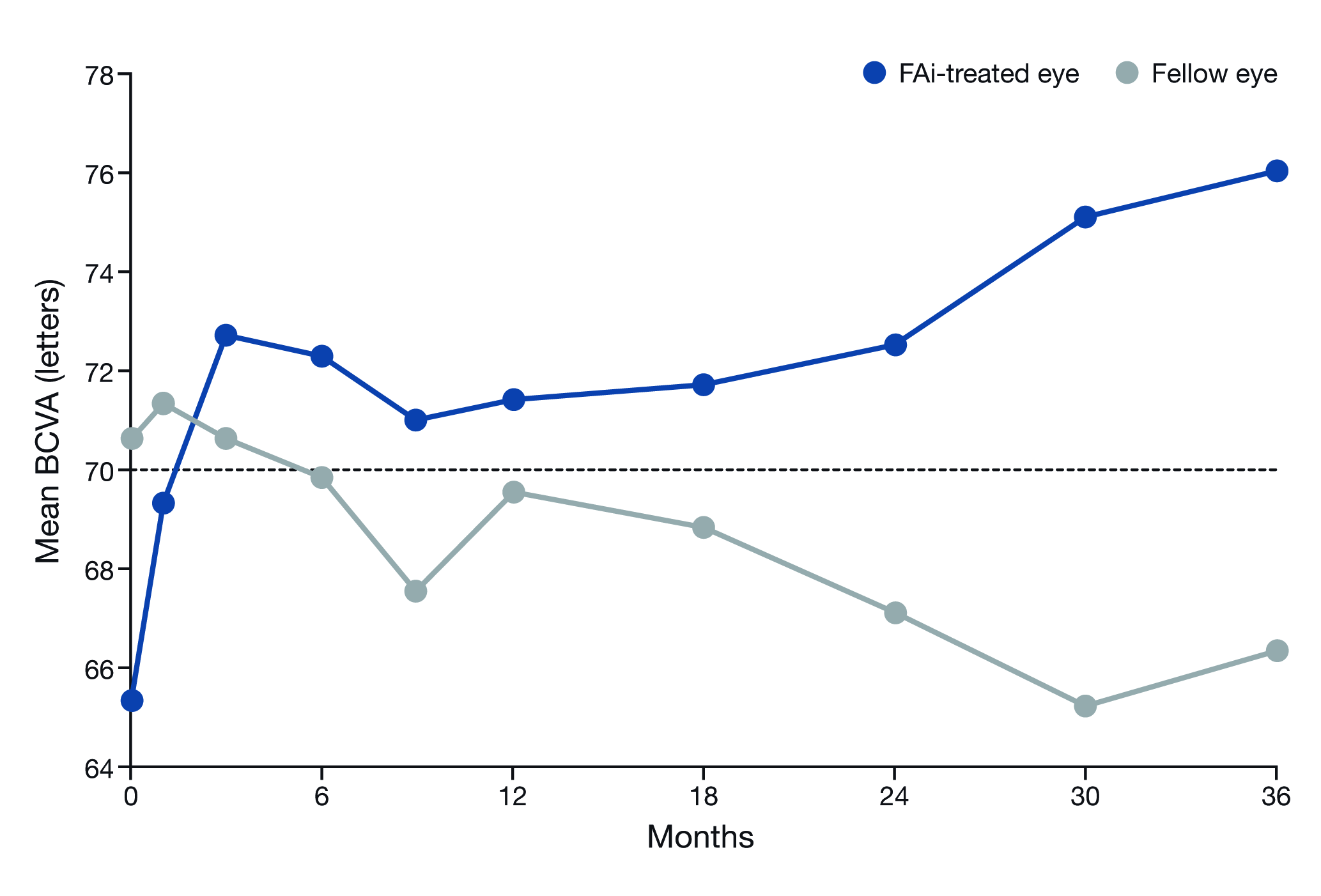

Supplement: Supplementary file 2 — Supplementary Figure 1. [file 41433_2021_1608_MOESM2_ESM.tif]

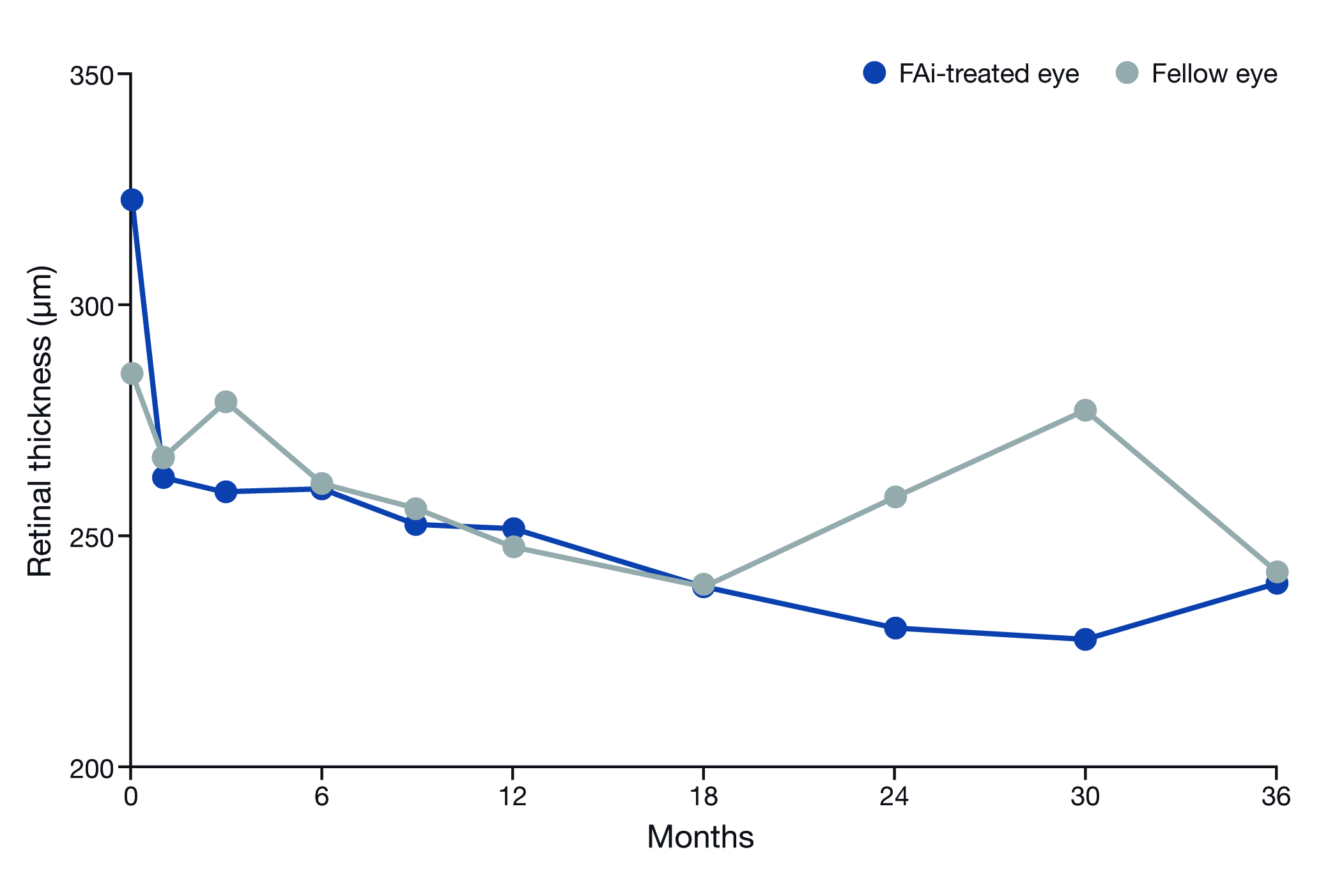

Supplement: Supplementary file 3 — Supplementary Figure 2 [file 41433_2021_1608_MOESM3_ESM.tif]
